# Centering decriminalization of suicide in low – and middle – income countries on effective suicide prevention strategies

**DOI:** 10.3389/fpsyt.2022.1034206

**Published:** 2022-11-17

**Authors:** Brenda K. Ochuku, Natalie E. Johnson, Tom L. Osborn, Christine M. Wasanga, David M. Ndetei

**Affiliations:** ^1^Shamiri Institute, Nairobi, Kenya; ^2^Department of Psychology, Kenyatta University, Nairobi, Kenya; ^3^Africa Mental Health Research and Training Foundation, Nairobi, Kenya; ^4^Department of Psychiatry, University of Nairobi, Nairobi, Kenya

**Keywords:** suicide decriminalization, suicide prevention, mental health, mental illness, LMICs, suicide psychopathology

## Abstract

Globally, over 800,000 people die by suicide every year. For every one completed suicide, 20 more attempts have been made. As previous attempts are one of the strongest predictors of future suicide, help-seeking in moments of crisis, particularly after an attempt, may have important implications for suicide prevention. Unfortunately, the criminalization of suicide in several countries hinders help-seeking, increases the stigmatization of those who attempt suicide and obstructs the accurate tracking of suicides. Here, we highlight the negative effects of suicide criminalization and discuss evidence-based strategies for suicide prevention such as means restriction, improved mental health literacy and access to psychosocial support, and responsible media coverage of suicides.

## Introduction

Nearly 800,000 lives are lost globally to suicide every year (1): that is 1 life every 40 seconds. Low– and middle–income countries (LMICs) account for 77% of these deaths (2). There are more people who die from suicide than malaria globally and deaths from suicide are comparable to those from HIV/AIDS (2). Yet, whereas public health funding and policy have predominantly focused on the prevention of these and other communicable diseases, spending and policy on suicide prevention lag behind (3).

Recent advances in research have enabled us to develop a better understanding of the complex experience of suicidality. Through these efforts, we have identified several factors that can contribute to an increased risk of suicide mortality. These include poor mental health, exposure to stressful life events such as the death of a loved one, financial problems, substance use disorders, and sex and sexuality (4, 5).

However, the most indicative factor for future suicide attempts is previous suicide attempts (2). This suggests that the period following an attempted suicide provides a crucial opportunity to intervene and prevent future attempts. Yet several countries — predominantly LMICs like Kenya — continue to criminalize suicide (6). This criminalization significantly hinders avenues to identify and provide help and support to individuals at risk of suicide mortality, who may seek to conceal their suicidality for fear of the legal implications.

In this perspective piece, we highlight that criminalizing suicide is not an effective deterrent to suicidality, leads to increased stigmatization of suicide, hinders efforts to better understand suicidality by weakening suicide surveillance, and serves as a major obstacle to the introduction of effective suicide prevention interventions. Whereas this perspective makes the case for and strongly encourages the countries that still criminalize suicide to decriminalize suicide, we center this call on effective strategies for the prevention of suicide. The goal of decriminalization is beyond the mere passing of legislation and must prioritize the implementation of strategies that effectively prevent suicide in both policy and practice.

## Attempted suicide as a crime: History, perspectives, and the case for decriminalization

The history of criminalizing suicide attempts is long and influenced by many factors including religion, politics, and colonization. Efforts to criminalize suicide were first informed by religious and spiritual perspectives. For example, the early writings of Saint Augustine presented the Christian commandment of “thou shall not kill” to include taking one's own life (7). This seems to have been the basis for why many predominantly Christian countries criminalized suicide (7). Similarly, in the Islamic tradition, suicide is viewed as a sin under Sharia Law, a code of conduct that informs the legal practices of many Islamic states (8).

Politically, suicide seems to have evolved from a crime against religion to a crime against the state. British Common Law delineated that one had no right to take their own life as it belonged to the state (6). Interestingly, the British Common Law informed the legal canon for many former British colonies, like Kenya, which found themselves criminalizing suicide even after British colonization ended (6).

Advances in scientific literature in the 19th and 20th centuries may have been the genesis of a shift in societal views and perspectives on suicide (9). During this period, cognitive and emotional distress were presented as complex phenomena that could also be caused by natural biological factors (9). Consequently, several countries repealed laws criminalizing suicide, predominantly in Europe and North America (10).

Advances in attitudes toward human rights, including the Convention on the Rights of Persons with Disabilities (11), have prompted many other countries to abolish laws that criminalize suicide. Furthermore, the World Health Organization Mental Health Action Plan 2020–2030 (12), which calls for human rights oriented policy to tackle suicide, has impelled many countries to commit to decriminalizing suicide.

Despite such breakthroughs, suicide remains criminalized in many countries around the world, particularly in LMICs in Africa and Southeast Asia which bear the biggest burden of suicide mortality (13–15). The law criminalizing suicide in Kenya, the 1930 Penal Code (16), was introduced and enacted by the British government. Although suicide was decriminalized in Great Britain in 1961, this change was not extended to its colonies including Kenya. In fact, post-colonial Kenya still upholds this British – enacted law that lists suicide as a misdemeanor punishable by imprisonment and/or a fine (16).

## What is the justification for the continued criminalization of suicide?

A common argument in support of criminalization is that it discourages people from attempting suicide. In other words, some argue that criminalization is a deterrent to suicide and as such, it may be an effective form of suicide prevention (17). But is this belief grounded in evidence? Literature suggests that this belief is misleading, and is at worst, erroneous. A recent study found no consistent evidence for lower suicide rates in countries that criminalize suicide compared to the global average (18). In fact, seven out of the 20 countries that criminalize suicide had higher rates than the global average and five out these seven were African countries (18). Moreover, studies that have looked at the rates of suicide post – decriminalization have found that in many countries, de-criminalizing suicide has not led to significant increases in suicide. For example, in Canada, New Zealand, and Ireland, when researchers compared the rates of suicide 10 years before and 10 years after suicide was decriminalized, they did not find a significant increase in suicide rates (19–21). Though there have been a few countries in which suicide rates have been reported to increase post- decriminalization, these findings have often been explained by more accurate and robust suicide surveillance and reporting post decriminalization rather than to decriminalization itself (10).

It seems that criminalizing suicide does not deter people from attempting suicide. What it does is complicate the lives and experiences of individuals at risk of suicide in many ways. We highlight three such ways.

The first is that criminalizing suicide leads to increased stigmatization and discrimination against individuals who attempt suicide. In Kenya, the national government issues a Certificate of Good Conduct that is essential for individuals to engage meaningfully in social, economic, and political life in Kenya. If one attempts suicide, they become a criminal and may fail to obtain this certificate (16). The consequence is that their path to meaningful societal engagement is limited. Added to that is the additional stigma of being a criminal (22, 23). This may lead some to utilize more lethal means to avoid the possibility of surviving a suicide attempt and possibly facing imprisonment and societal stigma (18).

The second is that criminalization of suicide limits the accuracy of suicide surveillance and reporting. Maintaining an accurate and robust report of suicides is undoubtedly important for policy, research, and practice. Such a record can inform important conversations on suicidality including those on suicide prevention. When suicide is criminalized, many people are not incentivized to accurately report suicide data. Deaths from suicide are sometimes recorded as “accidents” or “undetermined deaths” in order to avoid potential legal implications (10). In many countries, suicides often trigger police investigations which often involve long and tedious processes which many seek to avoid (10). Decriminalizing suicide would allow for more accurate tracking and surveillance of suicide thus facilitating important public health research, practice, and policy on suicidality.

Finally, criminalizing suicide is a significant barrier to help seeking. People at risk of suicide are likely also struggling with mental health problems, and already face significant barriers to help-seeking, especially in LMICs (24). Criminalizing suicide only exacerbates this for both individuals seeking help and caregivers seeking to support them. In Kenya, we have experienced firsthand how mental health practitioners often struggle to develop help-seeking avenues and protocols for individuals at risk of suicide or who attempt suicide due to the criminalization of suicide.

Aside from the human rights and political reasons to decriminalize suicide, the above-mentioned factors seem to confer that the advantages of criminalizing suicide are outweighed by the disadvantages. It is thus unsurprising that many countries around the world have decriminalized suicide. Yet in a few countries, especially LMICs in Africa and Southeast Asia, attempted suicide remains illegal (14, 15). We call for these holdout countries to decriminalize suicide.

## Centering decriminalization on effective suicide prevention strategies

There continues to be increased political and social pressure on the remaining countries where suicide is illegal to decriminalize it. As such, we believe that these countries will soon, or eventually, move to decriminalize suicide. But we fear that such changes may be “rubber stamp” or “token” efforts taken to change laws and policies that don't lead to direct changes in practice and reality for individuals at risk of suicide. As such, we seek to propose centering decriminalization on effective suicide prevention.

The primary reason for decriminalization is that it is an effective suicide prevention strategy. The single most important predictor of suicide is previous attempted suicide (2); some 40% of people who die by suicide have previously attempted (25). If we decriminalize suicide, then we open avenues for individuals who have attempted suicide to get help and increase chances of preventing subsequent suicide attempts. However, with the scarcity of appropriate and accessible mental health care services in most LMICs, it is essential that the decriminalization of suicide is accompanied with an increase in avenues for help and support to accommodate the anticipated increase in help-seeking behavior (18).

Decriminalization on its own merely provides the platform upon which effective suicide prevention strategies can be implemented. As such, it is important that as part of decriminalization, effective suicide prevention strategies are identified and made available.

### Suicide prevention strategies

#### Means restriction

Means restriction is an effective method of preventing suicide (26). Research on suicidality shows that one of the most significant factors predicting suicide mortality is access to a lethal means for suicide (26). A review of suicide methods found that the most common means of suicide are firearm-related deaths and deliberate self-poisoning (13). Firearm-related suicides are more common in high-income countries (HICs) (13). However, rates vary, with the United Kingdom and other European countries recording far less firearm suicides than the United States, which has much more lenient gun policy (13). In LMICs, a large proportion of suicide deaths are due to deliberate self-poisoning (13). In these countries, pesticides are often the most easily accessible poisons (13). Due to their high toxicity, those who attempt suicide by ingesting a pesticide often die as a result, particularly in settings where access to medical facilities is limited (27).

Thus, it seems that enforcing stricter regulations that limit access to lethal means may be an effective means of reducing suicides conducted in these methods. In HICs, this may be in the form of enforcing stricter laws on firearm purchase and possession. In LMICs, this may be regulatory action that limits access to frequently ingested toxic pesticides. Regulatory action replacing toxic pesticides in some countries with less toxic alternatives led to considerable reductions in suicides due to pesticide ingestion (27). Bans on frequently ingested pesticides also resulted in a decrease of pesticide suicides in five of the six countries studied, along with a decline in overall suicide rates in three countries (28). Sri Lanka saw a decrease in suicide rates by at least 93 000 deaths between 1995 and 2015 following bans on lethally poisonous pesticides (27). This evidence indicates that restriction of lethal means such as pesticides may be a promising avenue for reducing suicide mortality.

#### Improved mental health literacy and access to psychosocial support

There has been remarkable progress in advancing our understanding of mental health as well as human rights and the need to protect human rights. In addition, research has helped to elucidate potential causes, and risk and protective factors of suicide. That said, there is still a need to expand mental health literacy, particularly education on the association between poor mental health and suicide. These efforts should focus on communicating that suicide, as well as mental health conditions, are treatable and preventable. This messaging is especially important because mental health conditions, particularly mood disorders such as depression, are associated with 98% of suicides (4). Therefore, suicide can be reduced if people who are struggling with their mental health are aware of their treatment options and are able to access them easily.

#### Responsible media coverage of suicides

Media can be used as a tool for suicide prevention. However, it has often elicited the opposite effect. For instance, the use of language such as “committed” suicide in mainstream media acts to further stigmatize suicidality by furthering the narrative that it is criminal behavior.

Furthermore, suicides, particularly those of celebrities, are often sensationalized and glamorized by media outlets which can lead to “copycat suicides” (29). In the months after the suicide of popular comedian Robin Williams, there was a 10% rise in suicides in the United States (30). Of the 63 articles in the media which covered Robin's death, 27% romanticized his suicide and 46% provided details about the method (31). Furthermore, although his battle with mental illness and addiction was commonly featured, only 11% of the articles included information about help-seeking. This media coverage was linked to the increase of suicide mortality following Williams' death (31). Additionally, copies of suicide news articles have often been found near the body of the victim, further highlighting the important role of media coverage (29).

Explanations of this association are often framed around the social learning theory and propose that people learn the behavior of suicide by observing others deal with their life problems through suicide (29). Details about the method, such as the means that were used and where they were obtained from, can and often do strengthen this association. Responsible media coverage abstains from using terminology such as “committed” or “successful” suicide, doesn't offer too much detail about the method, and doesn't glamorize the suicide (29). Instead, the opportunity is taken to educate about mental health and to provide information about avenues for assistance such as helplines.

## Discussion

Suicide is a complex and important global problem. We have made great strides in our understanding of this issue, as well as mental health in general. It is time to translate theory to practice. If we are to mitigate suicide rates, research findings need to be complemented with effective policy and legislation in a timely manner. A first step is to repeal laws that criminalize suicide attempts in countries where it remains illegal. The criminalization of suicide is outdated and may cause more harm than good. Means restriction, improved mental health literacy and access to psychosocial support, as well as responsible media coverage are evidence-based suicide prevention methods. It is our hope that this brief enhances efforts to aid those who struggle with suicidality at the individual as well as the national and international level.

## Data availability statement

The original contributions presented in the study are included in the article/supplementary material, further inquiries can be directed to the corresponding author/s.

## Author contributions

All authors listed have made a substantial, direct, and intellectual contribution to the work and approved it for publication.

## Funding

This project was made possible through the support of a grant from Templeton World Charity Foundation, Inc. (Grant #TWCF0633).

## Conflict of interest

The authors declare that the research was conducted in the absence of any commercial or financial relationships that could be construed as a potential conflict of interest.

## Publisher's note

All claims expressed in this article are solely those of the authors and do not necessarily represent those of their affiliated organizations, or those of the publisher, the editors and the reviewers. Any product that may be evaluated in this article, or claim that may be made by its manufacturer, is not guaranteed or endorsed by the publisher.

## Author disclaimer

The opinions expressed in this publication are those of the author(s) and do not necessarily reflect the views of Templeton World Charity Foundation, Inc.
